# Biochemical Profile Variations Among Type 2 Diabetic Patients Stratified by Hemoglobin A1c Levels in a Saudi Cohort: A Retrospective Study

**DOI:** 10.3390/jcm14155324

**Published:** 2025-07-28

**Authors:** Abdulrahman Alshalani, Nada AlAhmari, Hajar A. Amin, Abdullah Aljedai, Hamood AlSudais

**Affiliations:** Department of Clinical Laboratory Sciences, College of Applied Medical Sciences, King Saud University, Riyadh 12372, Saudi Arabia

**Keywords:** liver enzymes, renal function, lipid profile, Saudi diabetic patients

## Abstract

**Background**: The global increase in type 2 diabetes mellitus (T2DM) cases necessitates the need for early detection of metabolic changes. This study investigated variations in liver enzymes, renal markers, electrolytes, and lipid profiles among T2DM patients stratified by hemoglobin A1c (HbA1c) categories to support early identification and better management of diabetes-related complications. **Methods**: A retrospective observational study at King Khalid University Hospital (KKUH), Riyadh, included 621 adult patients diagnosed with T2DM categorized into four HbA1c groups: normal (<5.7%), prediabetes (5.7–6.4%), controlled diabetes (6.5–7.9%), and uncontrolled diabetes (≥8.0%). Biochemical parameters included the liver profile: alkaline phosphatase (ALP) and bilirubin, renal profile: creatinine, blood urea nitrogen (BUN), glucose, sodium, and chloride, and lipid profile: cholesterol, high-density lipoprotein (HDL), low-density lipoprotein (LDL), and triglycerides. Regression models identified predictors of ALP, cholesterol, and LDL. **Results**: ALP was higher in uncontrolled diabetes (89.0 U/L, Q1–Q3: 106.3–72.0) than in the prediabetes group (75.0 U/L, Q1–Q3: 96.8–62.3). Sodium and chloride were lower in uncontrolled diabetes (Na: 138.3 mmol/L, Q1–Q3: 140.3–136.4; Cl: 101.1 mmol/L, Q1–Q3: 102.9–99.4) compared to the normal group (Na: 139.5 mmol/L, Q1–Q3: 142.4–136.9; Cl: 103.5 mmol/L, Q1–Q3: 106.1–101.7). LDL was lower in uncontrolled diabetes (2.1 mmol/L, Q1–Q3: 2.8–1.7) than in the normal group (2.8 mmol/L, Q1–Q3: 3.7–2.2), while triglycerides were higher in patients with uncontrolled diabetes compared to the normal group (1.45 mmol/L, Q1–Q3: 2.02–1.11 vs. 1.26 mmol/L, Q1–Q3: 1.44–0.94). Regression models showed low explanatory power (R^2^ = 2.1–7.3%), with weight, age, and sex as significant predictors of select biochemical markers. **Conclusions**: The study observed biochemical variations across HbA1c categories in T2DM patients, likely reflecting insulin resistance. Monitoring these markers in conjunction with HbA1c can enhance early detection and improve the management of complications.

## 1. Introduction

Type 2 diabetes mellitus (T2DM), characterized by insulin resistance and chronic hyperglycemia, accounts for approximately 90–95% of all diabetes cases and poses a significant global-health burden [1,2,3]. According to the International Diabetes Federation (IDF), the global prevalence of diabetes among adults aged 20–79 reached 10.5% in 2021 and is projected to increase by 46% by 2045, potentially affecting over 780 million individuals [4]. In Saudi Arabia, the situation is especially concerning. National data from 2016 to 2022 indicate a T2DM prevalence of 28% among adults [5], and projections estimate that, if unaddressed, more than half of the adult population could be affected by 2030 [6]. This escalating prevalence presents a critical challenge for the Saudi healthcare system, particularly due to the chronic complications and financial strain associated with diabetes care [7].

Early detection and diagnosis are critical for reducing diabetes-related complications and improving long-term outcomes [8]. Hemoglobin A1c (HbA1c) is a key diagnostic tool that reflects average blood-glucose levels over the previous two to three months, offering advantages over single-point glucose measurements [9]. HbA1c testing is recommended by major health organizations, including the American Diabetes Association (ADA), and is widely adopted due to its convenience and reliability. The ADA categorizes HbA1c as follows: normal (<5.7%), prediabetes (5.7–6.4%), controlled diabetes (6.5–7.9%), and uncontrolled diabetes (≥8.0%) [1]. In addition to its diagnostic value, HbA1c has been linked to subclinical cardiovascular dysfunction, even among individuals without diabetes, highlighting its broader relevance as a marker of metabolic and vascular health [10].

Chronic hyperglycemia in T2DM contributes to structural and functional damage across multiple organ systems, particularly the cardiovascular, hepatic, and renal systems [11]. Cardiovascular disease (CVD) remains a leading cause of morbidity and mortality among diabetic patients, driven by mechanisms such as endothelial dysfunction, chronic inflammation, and dyslipidemia [12,13]. In addition to CVD, diabetic patients exhibit elevated liver enzymes and are likely to acquire hepatic fat regardless of their body mass index (BMI) [14]. Nonalcoholic fatty liver disease (NAFLD) is frequently observed in this population and is associated with increased levels of alanine aminotransferase (ALT) and gamma-glutamyl transferase (GGT), reflecting hepatic fat accumulation [15]. These abnormalities are part of a broader metabolic dysfunction linked to insulin resistance and obesity [16]. Additionally, impaired renal function and chronic kidney disease (CKD) are highly prevalent in diabetic patients, with diabetic nephropathy contributing to electrolyte disturbances due to disrupted fluid and mineral regulation [17,18].

Given the increasing burden of T2DM and its systemic complications, early identification of biochemical alterations is essential for improving clinical outcomes. Biochemical markers including liver enzymes, renal function indicators, electrolytes, and lipid profiles are frequently affected by chronic hyperglycemia. However, limited data are available on how these parameters vary across different HbA1c categories in T2DM patients. This study was designed to evaluate these variations, with the aim of identifying metabolic patterns associated with glycemic control that may support early intervention and risk stratification in patients with T2DM. Based on this rationale, it is hypothesized that biochemical abnormalities increase progressively with worsening glycemic control, as reflected by rising HbA1c levels.

## 2. Materials and Methods

### 2.1. Study Design

This retrospective cross-sectional study was conducted at King Khalid University Hospital (KKUH) in Riyadh, Saudi Arabia, utilizing electronic health records to retrieve patient data. The study included adult patients (aged ≥ 18 years) who received care at KKUH between 1 January and 31 July 2024, had a diagnosis of T2DM, and had documented HbA1c levels along with comprehensive biochemical profiles. Patients were excluded if they were younger than 18 years of age, had missing or incomplete HbA1c or biochemical data, or had duplicate records. A total of 621 adult patients diagnosed with T2DM were included in this retrospective study, categorized into four groups based on HbA1c levels in accordance with the ADA standards: normal (<5.7%, n = 23), prediabetes (5.7–6.4%, n = 98), controlled diabetes (6.5–7.9%, n = 228), and uncontrolled diabetes (≥8.0%, n = 272). This classification facilitated an assessment of the relationship between glycemic status and biochemical parameters.

### 2.2. Patient Data

All biochemical analyses were performed retrospectively in a College of American Pathologists (CAP)—an accredited laboratory using standardized, quality-controlled assays, ensuring the reliability and comparability of the results. All biochemical assessments were performed using the Cobas^®^ 8100 automated analyzer to ensure accuracy and standardization.

The biochemical markers analyzed in this study encompassed liver function, renal function, lipid profiles, and glucose metabolism. Liver-function parameters included alkaline phosphatase (ALP), ALT, aspartate aminotransferase (AST), direct bilirubin, indirect bilirubin, total bilirubin, GGT, and total protein. Renal-function parameters included blood urea nitrogen (BUN), creatinine, osmolality, glucose, calcium, corrected calcium, phosphorus, potassium, sodium, chloride, carbon dioxide (CO_2_), and albumin. The lipid panel comprised total cholesterol, high-density lipoprotein (HDL), low-density lipoprotein (LDL), and triglycerides. Venous blood glucose concentrations were determined in the fasting state as part of the routine biochemical panel.

Demographic and clinical variables, including age, sex, BMI, height, weight, smoking status, and marital status, were obtained from electronic medical records to assess their potential associations with biochemical parameters. The presence of comorbidities, including CVD (operationally defined in this study based on diagnoses of circulatory complications and cardiomyopathy as documented in patient records), neuropathy, nephropathy, ketoacidosis, and musculoskeletal disorders, was recorded. However, detailed information regarding these specific comorbidities was not consistently documented in the medical-record system. Additionally, Metformin use was documented. All data were securely extracted and stored in Microsoft Excel for further statistical analysis. This study was approved by the Institutional Review Board (IRB) Sub-Committee for Health Sciences Colleges Research on Human Subjects at King Saud University—College of Medicine, Riyadh, Saudi Arabia (Approval No. E-24-8824; Approval Date: 22 May 2024).

### 2.3. Statistical Analysis

Statistical analyses were conducted using SPSS software (version 26). Graphical visualizations were generated using GraphPad Prism (version 10.4.1). Categorical variables were summarized as frequencies and percentages, while continuous variables were reported as medians and with first and third quartiles (Q1–Q3). Normality was assessed using the Shapiro–Wilk test. Comparisons of biochemical parameters across glycemic groups were conducted using one-way ANOVA for normally distributed variables and the Kruskal–Wallis test for non-normally distributed data. A *p* < 0.05 was considered statistically significant.

Correlation analyses were performed for variables with statistically significant differences across groups to explore relationships between biochemical parameters and demographic factors. Pearson’s correlation was used for associations between two continuous variables, Spearman’s correlation was applied for relationships between a continuous and a categorical variable, and point-biserial correlation was utilized for examining associations between a continuous and a dichotomous variable. Variables with a *p* < 0.2 in correlation analysis were included in multiple linear regression models, following the purposeful selection method to retain potentially relevant predictors [19]. Multicollinearity was assessed using the variance inflation factor (VIF), and no significant issues were detected. The strength of the models was evaluated using R-squared values to determine the proportion of variance explained by independent variables.

## 3. Results

### 3.1. Study Population

Table 1 presents the demographic and baseline characteristics across groups. The sex distribution across the study groups was approximately equal across groups, with a slightly higher proportion of females in the uncontrolled diabetes group (58.5%). The age distribution showed that adults over 60 years constituted the majority in every HbA1c category, with uncontrolled diabetes (57.4%), controlled diabetes (60.1%), pre-diabetes (61.2%), and normal (52.2%) levels. The median fasting glucose levels increased progressively across groups, from 5.9 mmol/L (Q1–Q3: 5.6–6.0) in the normal group to 9.4 mmol/L (Q1–Q3: 8.5–12.5) in the uncontrolled diabetes group. The prevalence of comorbidities was relatively low across all groups. The most common comorbidity was neuropathy, affecting 4.3% in the normal group, 3.1% in the prediabetes group, 0.9% in the controlled diabetes group, and 1.5% in the uncontrolled diabetes group. Lastly, Metformin use was most prevalent in the controlled diabetes group (6.1%) and least common in the normal group (4.3%).

### 3.2. Impact of HbA1c Levels on Liver-Function Parameters in Patients with T2DM

**Figure 1:**
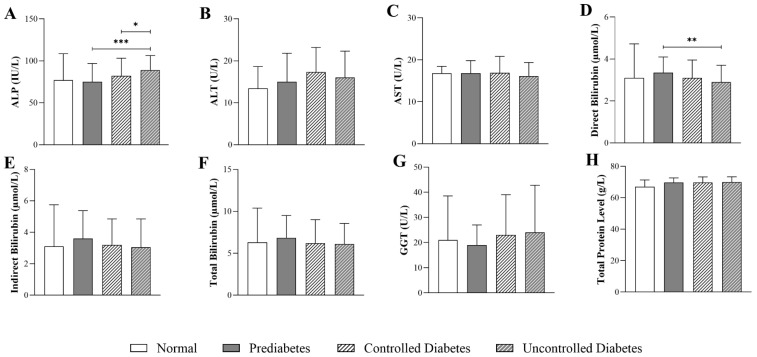
Differences in liver function parameters in patients with T2DM across HbA1c groups (normal: n = 23; prediabetes: n = 98; controlled diabetes: n = 228; uncontrolled diabetes: n = 272), including (**A**) ALP (alkaline phosphatase), (**B**) ALT (alanine aminotransferase), (**C**) AST (aspartate aminotransferase), (**D**) direct bilirubin, (**E**) indirect bilirubin, (**F**) total bilirubin, (**G**) GGT (gamma-glutamyl transferase), and (**H**) total protein levels. The statistical significance levels are indicated (* *p* < 0.05, ** *p* < 0.01, *** *p* < 0.001).

Figure 1 illustrates differences in liver-function parameters in patients with T2DM stratified by HbA1c levels. ALP levels were significantly higher in uncontrolled diabetes (89.0 U/L, Q1–Q3: 106.3–72.0) compared to prediabetes (75.0 U/L, Q1–Q3: 96.8–62.3) (*p* < 0.001). Direct bilirubin levels were significantly lower in the uncontrolled diabetes group (2.90 µmol/L, Q1–Q3: 3.70–2.00) compared to the prediabetes group (3.10 µmol/L, Q1–Q3: 3.95–2.20) (*p* < 0.01). No significant differences were observed in ALT, AST, indirect bilirubin, total bilirubin, GGT, and total protein levels across the glycemic categories.

### 3.3. Impact of HbA1c Levels on Kidney-Function Parameters in Patients with T2DM

**Figure 2:**
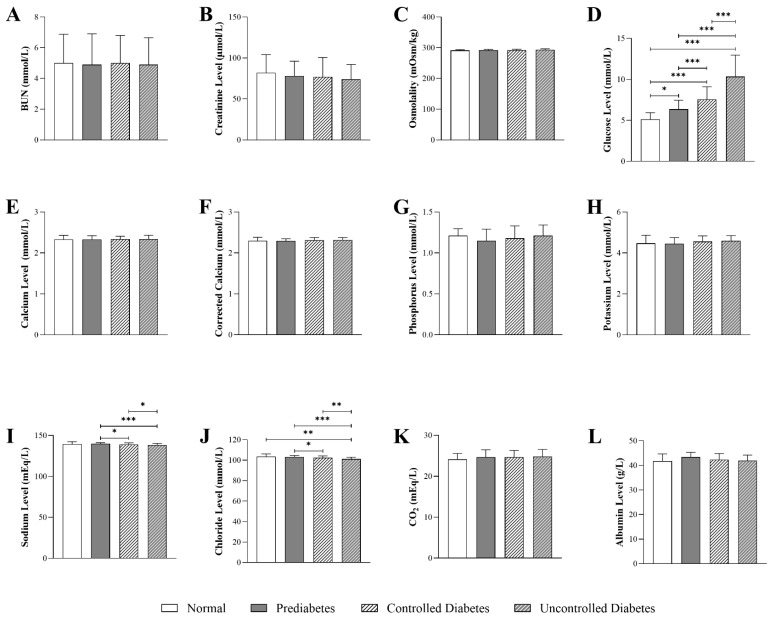
Differences in kidney function and electrolyte parameters in T2DM patients stratified by HbA1c levels: (normal: n = 23; prediabetes: n = 98; controlled diabetes: n = 228; uncontrolled diabetes: n = 272), including (**A**) BUN (blood urea nitrogen), (**B**) creatinine, (**C**) osmolality, (**D**) glucose, (**E**) calcium, (**F**) corrected calcium, (**G**) phosphorus, (**H**) potassium, (**I**) sodium, (**J**) chloride, (**K**) CO_2_ (carbon dioxide), and (**L**) albumin levels. The statistical significance levels are indicated (* *p* < 0.05, ** *p* < 0.01, *** *p* < 0.001).

Figure 2 shows that glucose levels were markedly elevated in the prediabetic (6.36 mmol/L, Q1–Q3: 7.45–5.58), controlled diabetic (7.56 mmol/L, Q1–Q3: 9.08–6.28), and uncontrolled diabetic groups (10.33 mmol/L, Q1–Q3: 12.97–7.84) compared to the normal group (5.13 mmol/L, Q1–Q3: 5.93–4.60) (*p* < 0.001). Sodium levels were significantly lower in the uncontrolled diabetic group (138.35 mmol/L, Q1–Q3: 140.37–136.40) compared to the normal group (139.50 mmol/L, Q1–Q3: 142.39–136.89) (*p* < 0.05). Similarly, chloride levels were significantly reduced in the uncontrolled diabetic group (101.17 mmol/L, Q1–Q3: 102.96–99.43) compared to the normal group (103.47mmol/L, Q1–Q3: 106.09–101.70) (*p* < 0.001). The prediabetic (103.20mmol/L, Q1–Q3: 104.69–101.00) and controlled diabetic groups (102.30mmol/L, Q1–Q3: 104.17–100.20) also exhibited lower chloride levels compared to the normal group (*p* < 0.01 and *p* < 0.05, respectively). No significant differences were observed across the glycemic categories in BUN, creatinine, osmolality, calcium, corrected calcium, phosphorus, potassium, and CO_2_ levels.

### 3.4. Impact of HbA1c Levels on Lipid Parameters in Patients with T2DM

**Figure 3:**
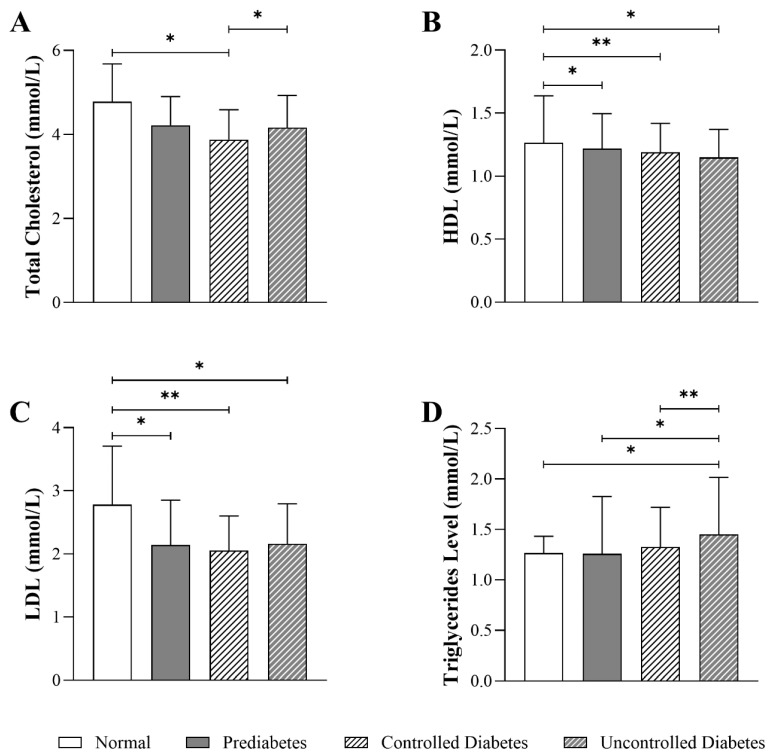
Differences in lipid profile parameters among T2DM patients across HbA1c groups (normal: n = 23; prediabetes: n = 98; controlled diabetes: n = 228; uncontrolled diabetes: n = 272), including (**A**) total cholesterol, (**B**) HDL (high-density lipoprotein), (**C**) LDL (low-density lipoprotein), and (**D**) triglycerides level. The statistical significance levels are indicated (* *p* < 0.05, ** *p* < 0.01).

Figure 3 shows that total cholesterol levels were significantly lower in the controlled diabetic group (3.87 mmol/L, Q1–Q3: 4.59–3.34) compared to the normal group (4.77 mmol/L, Q1–Q3: 5.68–3.26) (*p* < 0.05). However, the uncontrolled diabetic group (4.16 mmol/L, Q1–Q3: 4.93–3.59) showed no significant difference compared to the prediabetic and normal groups. HDL levels were lower in the uncontrolled diabetic (1.15 mmol/L, Q1–Q3: 1.37–1.01) and controlled diabetic groups (1.19 mmol/L, Q1–Q3: 1.42–1.01) compared to the normal group (1.26 mmol/L, Q1–Q3: 1.63–1.12) (*p* < 0.01). Similarly, patients in the prediabetic group had lower HDL levels (1.22 mmol/L, Q1–Q3: 1.50–1.05) than individuals in the normal group, but the difference was less pronounced. LDL levels were significantly lower in the uncontrolled diabetic group (2.16 mmol/L, Q1–Q3: 2.80–1.74) (*p* < 0.05), pre-diabetic group (2.14 mmol/L, Q1–Q3: 2.90–1.64) (*p* < 0.01), and controlled diabetic group (2.05 mmol/L, Q1–Q3: 2.60–1.64) (*p* < 0.01) compared with the normal group (2.78 mmol/L, Q1–Q3: 3.70–2.18). Triglyceride levels were significantly higher in the uncontrolled diabetic group (1.45 mmol/L, Q1–Q3: 2.02–1.11) compared to the normal group (1.26 mmol/L, Q1–Q3: 1.44–0.94) (*p* < 0.01). Similarly, the controlled diabetic group (1.32 mmol/L, Q1–Q3: 1.72–0.98) and the prediabetic group (1.26 mmol/L, Q1–Q3: 1.83–0.93) had higher triglyceride levels than the normal group (*p* < 0.05).

### 3.5. Relationship Between Demographic, Clinical Factors, and Biochemical Parameters in Diabetic Patients

To examine the relationship between demographic and clinical factors with biochemical parameters, correlation analyses were performed (Table A1). Variables demonstrating a p less than 0.2 were considered for further analysis in multiple regression models, while others were excluded due to there being fewer than three significant demographic associations. Based on this criterion, the selected independent variables for ALP were age (r = −0.057, *p* = 0.180), weight (r = 0.100, *p* = 0.028), comorbidity (r = 0.103, *p* = 0.016), and Metformin use (r = 0.078, *p* = 0.068). For total cholesterol, the selected variables were sex (r = 0.132, *p* = 0.003), age (r = −0.201, *p* < 0.001), and comorbidity (r = −0.066, *p* = 0.142). For LDL, the included variables were sex (r = 0.069, *p* = 0.123), age (r = −0.211, *p* < 0.001), and marital status (r = −0.058, *p* = 0.195). Variables exceeding the predefined significance threshold were excluded.

Table 2 shows that the regression models had low R^2^ values ranging from 2.1% to 7.3%, indicating weak explanatory power and limited ability to predict changes in ALP, cholesterol, and LDL levels. Although some predictors were statistically significant, these low R^2^ values suggest that additional, unmeasured factors may account for much of the variability in the biochemical parameters. However, some independent variables were statistically significant, suggesting they impact biochemical profiles. For ALP levels, weight was significantly and positively associated with ALP (B = 0.204, *p* = 0.047), indicating that for each unit increase in weight, ALP increased by 0.204 U/L. However, age (B = −2.093, *p* = 0.486), comorbidities (B = 6.406, *p* = 0.153), and Metformin use (B = 20.013, *p* = 0.096) were not significantly associated with ALP levels. The model explained 2.1% of the variance in ALP (R^2^ = 0.021, *p* = 0.042), suggesting a weak explanatory power but a statistically significant model. For cholesterol levels, both sex (B = 0.324, *p* < 0.001) and age (B = −0.264, *p* < 0.001) were significantly associated with cholesterol. Comorbidities (B = −0.240, *p* = 0.136) showed no significant effect. The model accounted for 7.3% of the variance in cholesterol (R^2^ = 0.073, *p* < 0.001), indicating a slightly more substantial, yet still weak, explanatory power. For LDL levels, both sex (B = 0.158, *p* = 0.046) and age (B = −0.235, *p* < 0.001) were significantly associated with LDL levels. Marital status (B = 0.056, *p* = 0.599) was not significantly related to LDL. The model explained 6.0% of the variance in LDL (R^2^ = 0.060, *p* < 0.001), suggesting a weak but statistically significant model.

## 4. Discussion

The international rise in T2DM cases demonstrates the need to discover early metabolic indicators associated with poor blood-sugar control. This retrospective analysis investigated the relationships between HbA1c ranges and biochemical markers, including liver enzymes, renal markers, electrolytes, and lipids, among Saudi adult patients diagnosed with T2DM. The rise in ALP across HbA1c categories was statistically significant, yet remained within the internationally accepted adult reference interval of 35–129 U/L, as defined by the International Federation of Clinical Chemistry and Laboratory Medicine (IFCC) standardized method [20]. A previous prospective cohort analysis, which included 132,000 hypertensive adults, reported the same pattern with ALP sat in the upper-normal band, not beyond the clinical limit [21]. Further support for these findings was provided in a study reporting notable increases in ALP, ALT, and AST in T2DM patients, which linked these alterations to hepatic insulin resistance and oxidative stress as the primary metabolic driver. Moreover, ALP is becoming acknowledged as an indicator of systemic metabolic stress, with elevated levels possibly signifying malfunction beyond the hepatobiliary system [22]. This perspective is supported by a study evaluating ALP levels in diabetic nephropathy and bone metabolism, which indicated that both diabetic patients with and without nephropathy exhibit elevated ALP levels [23]. Although our findings demonstrated a significant positive association between ALP and HbA1c levels, several studies have reported contrasting results, showing either weak or non-significant associations between ALP levels and glycemic status [24,25]. This may be influenced by certain limitations of our current study, including the lack of detailed medication usage and clinical presentations.

The current study observed an inverse relationship between HbA1c and total cholesterol levels in patients with T2DM. This result aligns with a 2025 dietary intervention trial, which indicated that subjects exhibiting enhanced glycemic control also had a notable reduction in total cholesterol levels, hence strengthening the correlation between glucose regulation and lipid balance [26]. Furthermore, findings from a 52-week randomized trial showed that nutritional treatment caused a reduction in total cholesterol levels in T2DM patients, suggesting that total cholesterol variation follows metabolic changes [27]. However, this trend contrasts with several cohort studies that have indicated a strong association between HbA1c and elevated total cholesterol levels, particularly in individuals with poor glycemic control [28,29,30,31].

In addition, our findings indicate an inverse relationship between HbA1c and LDL in patients with T2DM, with significantly lower LDL concentrations in all diabetic groups compared to the normal group. The results aligned with previous research showing reduced or unchanged LDL concentrations in diabetic patients, particularly in those undergoing lipid-lowering treatment when HbA1c remains persistently elevated [32,33]. A previous study conducted in Saudi Arabia found that total cholesterol and LDL levels in diabetic patients do not have a significant statistical relationship with HbA1c, but may be explained by population or treatment factors. However, this trend contrasts with other studies that show that DM patients who poorly manage blood-glucose levels demonstrate elevated LDL concentrations alongside atherogenic lipid patterns [21,31,34,35,36].

Within HbA1c categories, the observed biochemical alterations in our cohort reflect the activation of hepatic, lipid metabolic, and renal osmotic pathways associated with decreasing glycemic control, providing a foundation for the consequent discussion. The persistent increase of ALP in diabetic cohorts, reaching statistical significance, suggests hepatic insulin resistance and chronic systemic inflammation, corroborating previous findings that associate ALP with vascular calcification, nephropathy, and bone metabolism in T2DM [21,22,23,37]. Although direct bilirubin was not an independent predictor in our multivariate model, its consistent decrease across higher HbA1c categories is significant. Reduced bilirubin levels have been linked to lower antioxidant defenses and an increased risk of T2DM and NAFLD, indicating that the observed decline may signify escalating oxidative stress as glycemic management decreases [38,39,40]. In addition, patients with uncontrolled diabetes experiencing hyponatremia and hypochloremia match the conditions associated with osmotic diuresis and proximal tubular dysfunction that often precedes clinical nephropathy as possible signs of early renal impairment [18,41].

Patients with uncontrolled diabetes demonstrated a dyslipidemia pattern that includes diminished HDL levels together with elevated triglycerides as strong markers for the atherogenic lipid phenotype and increased cardiovascular risks [31,32,35,36]. Notably, the results revealed unexpectedly reduced LDL levels in diabetic groups, potentially due to unidentified factors, including the use of cholesterol medications and variations in LDL particle measures not evaluated by routine lipid assays [33,42]. Moreover, the inverse relationship between HbA1c and total cholesterol, although inconsistent with findings from several population-based studies, may indicate the impact of simultaneous lifestyle changes or pharmacological interventions aimed at both glycemic management and lipid control [26,27].

This study has several limitations. First, the cross-sectional retrospective design prevents the establishment of causal relationships between glycemic status and biochemical alterations. Second, the unequal sample sizes among HbA1c categories may have reduced statistical power and widened confidence intervals, potentially limiting the ability to detect actual differences. In particular, the relatively small sample size of the normal HbA1c group (n = 23) compared to the larger diabetic subgroups may have limited the statistical power of intergroup comparisons and should be considered when interpreting the results. Third, the dataset lacked important confounding variables such as dietary intake, physical activity, duration of T2DM, specific details about comorbidities and socioeconomic status, which could influence both glycemic control and metabolic profiles. Fourth, data on medications such as insulin, SGLT2 inhibitors, statins, and diuretics were incomplete and therefore not included in the analysis. This represents a potential confounding factor, as these agents may significantly affect biochemical parameters. Fifth, smoking history may have been underreported due to reliance on electronic records. Lastly, the study was conducted at a single tertiary care center in Saudi Arabia, which may limit the generalizability of the findings to broader or more diverse populations.

Future research should employ long-term prospective designs to determine the relationship between the increase in HbA1c values and biochemical changes, particularly during the early stages of impaired glycemic control. Subsequent research should proactively gather comprehensive medication histories, encompassing all glucose-lowering and lipid-modifying medications, to distinguish pharmacological effects from underlying biochemical alterations. Furthermore, sociodemographic factors, including dietary patterns and exercise habits, along with income status, require assessment for effective management of variables affecting glycemic control and biochemical markers. Finally, differentiating between disease-related symptoms and treatment responses depends on proper medical therapy data, including DM and cholesterol medications. These additional efforts would offer a more whole basis for knowledge of the glycemic influence on systemic metabolism and direct early, specific treatments in clinical practice.

## 5. Conclusions

In summary, this study assessed biochemical variances among HbA1c categories in a Saudi adult population with T2DM, applying a retrospective dataset. The results showed that ALP and direct bilirubin levels increased in all cohorts with a HgbA1c level of ≥5.7% compared to the normal group with a level of <5.7%. Sodium and chloride levels were reduced in the uncontrolled diabetes cohort within renal and electrolyte parameters. The diabetic groups indicated reduced LDL and HDL levels, alongside elevated triglyceride levels, while total cholesterol was decreased in prediabetic and controlled diabetic individuals. However, due to the cross-sectional design and weak model performance, further prospective studies are necessary before clinical translation. The findings indicate variations in metabolic markers across different glycemic categories and suggest the potential value of further analyses to more accurately define these associations, especially by applying larger datasets and longitudinal study designs.

## Figures and Tables

**Table 1:** Characteristics of T2DM patients included in the study stratified by HbA1C levels.

Variable	Groups			
	Normal (<5.7%) (23 Patients)	Prediabetes (5.7–6.4%) (98 Patients)	Controlled Diabetes (6.5–7.9%) (228 Patients)	Uncontrolled Diabetes (≥8.0%) (272 Patients)
**Sex**, n (%)				
Male	11 (47.8)	46 (46.9)	110 (48.2)	113 (41.5)
Female	12 (52.2)	52 (53.1)	128 (51.8)	159 (58.5)
**Age** (years), n (%)				
18–30	0 (0)	0 (0)	4 (1.8)	12 (4.4)
31–40	1 (4.3)	1 (1.0)	7 (3.1)	4 (1.5)
41–50	2 (8.7)	11 (11.2)	29 (12.7)	35 (12.9)
51–60	8 (34.8)	26 (26.5)	51 (22.4)	64 (23.5)
>60	12 (52.2)	60 (61.2)	137 (60.1)	156 (57.4)
**Glucose fasting** (mmol/L), median (Q1–Q3)	5.9 (5.6–6.0)	6.7 (6.1–7.3)	8.4 (7.1–9.2)	9.4 (8.5–12.5)
**BMI**, median (Q1–Q3)	32.8 (26.2–38.9)	29.2 (26.1–32.0)	30.1 (26.3–33.6)	30.1 (26.8–33.4)
**Height** (cm), median (Q1–Q3)	157.0 (152.3–165.5)	161.0 (156.0–170.0)	162.0 (152.0–169.0)	159.0 (153.0–168.0)
**Weight** (kg), median (Q1–Q3)	80.5 (64.5–94.3)	77.0 (63.2–86.8)	77.0 (64.0–89.0)	78.0 (67.1–87.5)
**Smoking Status**, n (%)				
Smoker	0 (0.0)	2 (2.0)	3 (1.3)	0 (0.0)
Non-smoker	23 (100.0)	96 (98.0)	225 (98.7)	272 (100.0)
**Marital Status**, n (%)				
Single	5 (21.7)	19 (19.4)	55 (24.1)	63 (23.2)
Married	18 (78.3)	79 (80.6)	173 (75.9)	209 (76.8)
**Comorbidity**, n (%)				
Circulatory Complications	0 (0.0)	1 (1.0)	0 (0.0)	1 (0.4)
Cardiomyopathy	0 (0.0)	0 (0.0)	1 (0.4)	0 (0.0)
Neuropathy	1 (4.3)	3 (3.1)	2 (0.9)	4 (1.5)
Nephropathy	0 (0.0)	0 (0.0)	1 (0.4)	0 (0.0)
Ketoacidosis	0 (0.0)	0 (0.0)	1 (0.4)	0 (0.0)
Musculoskeletal	0 (0.0)	0 (0.0)	0 (0.0)	1 (0.4)
Insulin resistance	2 (8.7)	0 (0.0)	1 (0.4)	0 (0.0)
No comorbidity	20 (87.0)	94 (95.9)	221 (96.9)	265 (97.4)
**Taking Metformin**, n (%)				
No	22 (95.7)	90 (91.8)	214 (93.9)	265 (97.4)
Yes	1 (4.3)	8 (8.2)	14 (6.1)	7 (2.6)

BMI: Body mass index.

**Table 2:** Multivariate regression models assessed the relationships between the independent variables (sex, age, weight, marital status, comorbidities, and Metformin use) and the biochemical parameters (ALP, cholesterol, and LDL levels).

Independent Variables	Coefficient (B)	R Square
	**ALP**	0.021 (*p* = 0.042)
Age	−2.093 (*p* = 0.486)	
Weight	0.204 (*p* = 0.047)	
Comorbidity	6.406 (*p* = 0.153)	
Metformin	20.013 (*p* = 0.096)	
	**Cholesterol**	
Sex	0.324 (*p* ≤0. 001)	
Age	−0.264 (*p* ≤ 0.001)	0.073 (*p* ≤ 0.001)
Comorbidity	−0.240 (*p* = 0.136)	
	**LDL**	
Sex	0.158 (*p* = 0.046)	
Age	−0.235 (*p* ≤ 0.001)	0.060 (*p* ≤ 0.001)
Marital Status	0.056 (*p* = 0.599)	

ALP: alkaline phosphatase, LDL: low-density lipoprotein.

## Data Availability

The data presented in this study are available on request from the corresponding author.

## Abbreviations

The following abbreviations are used in this manuscript:

ADA	American Diabetes Association
ALP	Alkaline Phosphatase
ALT	Alanine Aminotransaminase
AST	Aspartate Aminotransferase
BMI	Body Mass Index
BUN	Blood Urea Nitrogen
CAP	College of American Pathologists
CKD	Chronic Kidney Disease
CO_2_	Carbon Dioxide
CVD	Cardiovascular Disease
DM	Diabetes Mellitus
GGT	Gamma-Glutamyl Transferase
HbA1c	Hemoglobin A1c
HDL	High-Density Lipoprotein
IDF	International Diabetes Federation
IRB	Institutional Review Board
KKUH	King Khalid University Hospital
LDL	Low-Density Lipoprotein
NAFLD	Nonalcoholic Fatty Fiver Disease
Q1–Q3	First and third quartiles
T2DM	Type 2 Diabetes Mellitus

## Appendix A

**Table A1:** The correlation model.

Independent Variables	Dependent Variables							
	ALP	Direct Bilirubin	Chloride	Glucose Level	Sodium Level	Total Cholesterol	LDL	Triglycerides Level
**Sex**	0.052 (*p* = 0.221)	−0.040 (*p* = 0.353)	0.026 (*p* = 0.533)	0.040 (*p* = 0.333)	0.010 (*p* = 0.814)	0.132 (*p* = 0.003)	0.069 (*p* = 0.123)	−0.038 (*p* = 0.400)
**Age**	−0.057 (*p* = 0.180)	0.065 (*p* = 0.132)	−0.045 (*p* = 0.274)	−0.044 (*p* =0.288)	−0.038 (*p* =0.364)	−0.201 (*p* ≤0.001)	−0.211 (*p* ≤0.001)	0.023 (*p* =0.608)
**BMI**	0.037 (*p* = 0.458)	−0.019 (*p* = 0.700)	0.005 (*p* = 0.914)	0.060 (*p* = 0.214)	−0.045 (*p* = 0.356)	0.039 (*p* = 0.438)	0.053 (*p* = 0.292)	0.034 (*p* = 0.499)
**Height**	0.024 (*p* = 0.615)	0.021 (*p* = 0.662)	−0.001 (*p* = 0.984)	−0.050 (*p* = 0.288)	0.055 (*p* = 0.240)	−0.053 (*p* = 0.285)	−0.055 (*p* = 0.266)	0.070 (*p* = 0.160)
**Weight**	0.100 (*p* = 0.028)	−0.010 (*p* = 0.829)	0.031 (*p* = 0.483)	0.049 (*p* = 0.262)	−0.005 (*p* = 0.912)	0.039 (*p* = 0.410)	0.019 (*p* = 0.678)	0.146 (*p* = 0.002)
**Smoking Status**	−0.019 (*p* = 0.657)	0.010 (*P* = 0.819)	0.005 (*p* = 0.907)	−0.011 (*p* = 0.795)	0.024 (*p* = 0.558)	−0.054 (*p* = 0.226)	−0.044 (*p* = 0.322)	−0.017 (*p* = 0.697)
**Marital Status**	−0.038 (*p* = 0.368)	0.040 (*p* = 0.346)	0.071 (*p* = 0.085)	−0.046 (*p* = 0.268)	0.018 (*p* = 0.667)	−0.030 (*p* = 0.495)	−0.058 (*p* = 0.195)	−0.019 (*p* = 0.674)
**Comorbidity**	0.103 (*p* = 0.016)	0.000 (*p* = 0.994)	0.059 (*p* = 0.155)	0.010 (*p* = 0.802)	−0.037 (*p* = 0.375)	−0.066 (*p* = 0.142)	−0.044 (*p* = 0.325)	−0.033 (*p* = 0.456)
**Metformin**	0.078 (*p* = 0.068)	−0.004 (*p* = 0.932)	−0.009 (*p* = 0.826)	0.014 (*p* = 0.729)	−0.110 (*p* = 0.008)	−0.007 (*p* = 0.874)	0.016 (*p* = 0.722)	−0.018 (*p* = 0.687)

BMI; Body mass index, ALP: alkaline phosphatase, LDL: low-density lipoprotein.
